# The Integrated Electronic Health System in Iranian health centers: benefits and challenges

**DOI:** 10.1186/s12875-023-02011-x

**Published:** 2023-02-18

**Authors:** Sasan Ghorbani Kalkhajeh, Azam Aghajari, Behnaz Dindamal, Zohreh Shahvali-Kuhshuri, Farzad Faraji-Khiavi

**Affiliations:** 1 Healthcare Services Management, Department of Public Health, School of Health, Abadan University of Medical Sciences, Abadan, Iran; 2 Department of Health Services Management, School of Health, Ahvaz Jundishapur University of Medical Sciences, Ahvaz, Iran; 3 Department of Health Services Management, School of Health, Student Research Committee, Ahvaz Jundishapur University of Medical Sciences, Ahvaz, Iran; 4 Social Determinants of Health Research Center, Ahvaz Jundishapur University of Medical Sciences, Ahvaz, Iran

**Keywords:** The Integrated Electronic Health System, Health center, Electronic health record

## Abstract

**Background:**

Electronic Health Records (EHRs) were introduced to all Iranian medical universities in 2015 with the launch of Integrated Electronic Health System (which is known as SIB: a Persian backronym in Persian meaning *apple*), and a number of studies were conducted on SIB. However, most of these studies did not consider the benefits and challenges of adopting SIB in Iran. Therefore, the present study aimed to identify the benefits and challenges of SIB in health centers of Khuzestan Province, Iran.

**Methods:**

This was a qualitative study using qualitative conventional content analysis conducted on 6 experts and 24 users of SIB in six health centers of three cities in Khuzestan province, Iran. The participants were selected using a purposeful sampling method. Maximum variation was considered in selecting the group of users, and snowball sampling was used in the group of experts. Data collection tool was semi-structured interview. Data analysis was performed using thematic analysis.

**Results:**

Overall, 42 components (24 for benefits and 18 for challenges) were extracted from the interviews. Common sub-themes and themes were identified for challenges and benefits. The components formed 12 sub-themes, and they were placed in 3 main themes, namely structure, process and outcome. 1) Structure included four sub-themes of Financial resources, Human resources, Facilities, and Access to the Internet; 2) Process involved three sub-themes of Training, Providing services, and Time and workload; and 3) Outcome incorporated five sub-themes of Quality of health services, Access, Safety and personal distance, Screening and evaluation, and Research.

**Conclusions:**

In the present study, the benefits and challenges of adopting SIB were examined in three themes: structure, process, and outcome. Most of the identified benefits were related to the theme of outcome, and most of the identified challenges were related to the theme of structure. Based on the identified factors, by strengthening the benefits of SIB and also trying to eliminate or reduce its challenges, it is possible to institutionalize and use it more effectively in order to solve health problems.

## Background

In 2019, the World Health Organization defined e-health as the cost-effective and safe use of information and communication technologies to support health and health-related fields [1]. Given the rapid development of information technology and the tremendous potential of Electronic Health Records (EHRs), it seems these electronic systems will be the predominant form of health record systems and the basis for all patient-related communications in the near future [2]. EHR is a collection of information related to the health of citizens, from birth to death, which is stored continuously and electronically over time [3–5].

Various studies have acknowledged the benefits of EHR. These benefits include improved health care quality [6–8], customer satisfaction [9], on-time access to data [10], access to diverse clinical data [11], excellent capacity for storing and retrieving medical history, enhanced efficiency of healthcare systems [12], cost reductions, fewer medication errors, better data accessibility and tracking, and improved clinical outcomes [13]. Despite all their merits, the challenges associated with the adoption and application of EHRs have been reported in the literature [14]. These challenges are related to the integrity and availability of healthcare data and the possible risks for patient safety [15], information privacy [16], user resistance, poor technological knowledge, and insufficient computer skills, which may widen the gap between developing and developed countries [17, 18].

Secginli et al. assessed health professionals’ attitudes towards EHRs in primary health care settings in Turkey. Most of their respondents were satisfied with EHRs and agreed with it benefits but were against the barriers. However, the majority of the respondents agreed that EHRs are costly, need frequent revisions, and are frequently down [19]. In Ireland, the majority of the physicians and nurses agreed that EHR implementation improved patient care and safety, communication, and the legibility and clarity of patient care orders [20].

According to various studies in developed countries such as the United States, 13% of primary health care centers used EHRs in 2000, which increased to 49% in 2007 [21]. However, developing countries are at a disadvantage compared with developed countries due to their own challenges such as lack of technological, cultural, organizational, and legal infrastructure and the existence of human obstacles [22]. Developing countries, including Iran, are no exception when it comes to the developments related to the creation of EHR. In Iran, Integrated Electronic Health System (which is known as SIB: a Persian backronym in Persian meaning *apple*) was proposed by the Health Deputy of the Iranian Health Ministry for this purpose. It was put into operation in February 2015 [23], and to date, EHRs have been created for more than 73 million people in this system [24]. SIB has been launched in more than 36,000 urban and rural areas, with more than 130,000 healthcare personnel working with it [25]. Prior to SIB implementation, several electronic health record programs were tried in primary health care centers, all of which were rejected due to a myriad of factors [26]. Among the projects implemented in this field, SIB can be considered the most complete and up-to-date system because in addition to being online, it can connect to an integrated network that has a nationwide coverage [3].

All information related to households along with the type of health services required in community health centers is recorded in SIB. Some of the goals of SIB are: issuing EHRs for the public, creating a national database of health, providing integrated health services to the Iranian population throughout the country (especially low-income areas to increase social justice), providing health services based on specific needs of age groups, and establishing a referral system [3]. The most important functions of SIB include registration of people, registration of events, provision of health indicators, primary screening of diseases, immunization and vaccination system, geriatric care, pregnancy care, identification of risk factors of non-communicable diseases such as cardiovascular diseases, diabetes, cancers, strokes, and the mental health care system. According to the goals and general performance of SIB, the most important and common services are offered in the field of primary health care [24].

Due to the huge costs of setting up and using information systems, their incorrect selection and failure to address their weaknesses can lead to their failure [27]. Implementation and internalization of SIB were accompanied with ambiguities and lack of clarifications because the system was designed to meet the needs of all groups of clients, healthcare providers, managers, and screening experts of health programs, as well as policy makers and researchers. Despite the importance of SIB in health management of people, its design and implementation has also faced many challenges. One of the important challenges is that its performance and capabilities are to a large extent affected by the level of acceptance and satisfaction of users [23]. Kabir et al. assessed the satisfaction of users to be below the average level [23]. The results of Mohammadi Abnavi and Saeed's research showed that the quality of vaccination registration and its reporting was the strongest aspect of SIB while the performance of user entry and exit was the weakest [24]. Naqibzadeh and Safari also proposed solutions to strengthen SIB using the experience of users, which include items such as improving the reporting mechanism, providing alerts to perform vital and urgent operations regarding children and women, and identifying babies who whose follow-up care is due or overdue [28].

SIB was designed as the main information basis for health systems and is currently implemented all over the country; hence any problem in the performance of this system can affect the whole health system function [23]. Since the results of studies conducted on EHR in developed countries are not necessarily applicable in developing countries, it is necessary to conduct independent research in countries like Iran. If sufficient studies are not done, the challenges of these systems will not be identified, bringing about disruption in the service provision process and dissatisfaction of users and clients. Studies on SIB are more focused on topics such as the degree of the realization of its outcomes [26], evaluation of its usability [29], evaluation of its success [25], measurement of satisfaction with it [23] and evaluation of the level of acceptance of users [30, 31], and very few studies have investigated the challenges and benefits of SIB. The results of these few studies also indicate that more research is still needed on various dimensions of SIB in Iran, namely the structure, the process, and the outcome. To the best of our knowledge, no study has yet investigated the challenges and benefits of SIB based on the three items of structure, process, and outcome. Given the importance of achieving the goals and applications of SIB as well as the role of SIB in health information management in Iran and its effect on the quality of the service delivery process, a study addressing these dimensions will provide a deeper understanding of the subject. Therefore, this qualitative study was conducted to identify the challenges and benefits of SIB through a detailed examination of the views of SIB users and experts. By highlighting its strengths and proposing solutions to its challenges, the results of this study can provide the basis for improving the capabilities of SIB and making it more efficient so that it can be successfully implemented and provide better services. The qualitative method adopted in this study contributes to a better understanding of the data and people's points of view and making more valid conclusions from them in this regard. The present qualitative study aimed to identify the benefits and challenges of SIB in health centers of three cities in Khuzestan Province, Iran.

## Methods

This section was prepared based on consolidated criteria for reporting qualitative research(COREQ) guidelines.

### Study design

#### Theoretical framework

This was a qualitative study using qualitative conventional content analysis carried out in 2020. It was aimed to identify the benefits and challenges of the Integrated Electronic Health System (aka SIB) in Iran. Qualitative content analysis is a research method for subjectively interpreting the content of qualitative data through a systematic classification process of coding and identifying themes [32].

#### Participants and sampling

The research population included experts, health care providers, and physicians using SIB, and the research sample involved 30 of these individuals. Participants of this study were selected from two main groups of users and experts. Three doctors and 21 health care providers (from different units in health centers) were the SIB users, and a group of six experts consisting of one system leader, three evaluation experts, one network development unit expert and one Information Technology (IT) expert were the SIB experts. The inclusion criteria for users was to have at least one year of experience in health centers and familiarity with SIB (at least one year of work experience with this system). The inclusion criteria for experts was at least one year of experience in the related profession.

The participants were selected through purposeful sampling. Maximum variation was considered in selecting the users group, and snowball sampling was used in the experts group. In order to increase the comprehensiveness of the research, the researchers interviewed individuals at different access levels and system users in different units of health centers. For this purpose, an attempt was made to include experts from different specialties related to SIB including evaluation, network development, and IT. On the other hand, users were different based on their position and field of education. Service providers were also selected from different units such as family health, nutrition, mental health, and midwifery. The interviews were conducted face-to-face. In this study, none of the participants wanted to withdraw from the study, and all selected individuals participated in the interviews.

#### Setting

Participants were selected from six health centers located in three cities (Ahvaz, Shoushtar & Omidiyeh) of Khuzestan province, Iran. These health centers are affiliated to Ahvaz Jundishapur University of Medical Sciences and provide primary health care services (such as family health, health education, nutrition, mental health, etc.) and play a significant role in facilitating the access of people living in cities to primary care and maintaining and improving health in these areas. SIB is implemented in these centers for electronic documentation of all services, identification and resolution of the health problems of the population covered, and management of large volumes of information. These three cities were the first in the province in which SIB was implemented. During the interviews, no other person was present except the interviewers and the participants.

#### Data collection

Semi-structured interviews including open-ended questions were designed to gather the respondents' views about SIB. The respondents were interviewed individually and face-to-face and were asked to provide their views about two primary questions: 1. What are the challenges of adopting SIB in health centers? 2. What are the benefits of adopting SIB in health centers?

Then based on the internal issues related to the system, the respondents were directed toward problems of different parts of SIB. Interviews lasted from 40 to 60 min depending on the respondents' time and willingness. Data collection continued until data saturation. Saturation is the most common principle for determining the adequacy of samples in qualitative studies, and achieving saturation has become an important and fundamental component in this type of research, which makes the data collection process valid. In “data saturation”, saturation is the point at which no more concepts or insights are identified. This means that the data starts to iterate, making more data collection redundant and indicating that the adequate sample size is obtained [33]. In the current study, saturation was judged when no new themes or information were identified from the interviews, thus the sampling and data collection process was stopped after last 3 informants did not provide new information.

The interviews were conducted by the second and third researchers who were MSc. students of health management and had prior experience conducting similar interviews. Both interviewers were female. None of the researchers were working in the mentioned health centers, and the interviewers introduced themselves to the research sites by obtaining a letter of introduction from the Research Deputy of Ahvaz Jundishapur University of Medical Sciences. The time and place of the interviews were agreed upon at the interviewees’ convenience. They agreed to be later called for further clarification. All interviews were recorded using a tape recorder, carefully listened to, and transcribed verbatim. An attempt was made to conduct the interviews without bias and to write only the whole content. The transcriptions of the interviews were provided to the participants to confirm their accuracy (member check) and they gave feedback on the transcripts. Negative/discrepant results were addressed and parts that did not express their views were corrected.

### Data analysis

Thematic analysis was used to data analysis. In this study, data familiarization was achieved by writing interviews, reading the transcripts, and re-listening the recorded interviews. Then, the texts were coded and summarized. Two different coders coded the data on two separate occasions, and then the codes were compared, and conflicts were addressed. In this research, the main themes were already known, and after coding the initial interviews, subthemes were formed. The text was indexed using codes related to the themes and sub-themes of the conceptual framework. A chart was used to view all data. In the final step, the relationships between the concepts and the data of the charts were interpreted.

### Ethical issues

The study started after obtaining approval from the Ethics Committee of Ahvaz Jundishapur University of Medical Sciences (Ref. ID.: IR.AJUMS.REC.1396.783). In addition, prior to commencement of the interview sessions, informed consent was obtained from the participants. The participants were clearly briefed on their right to withdraw from the study at any time even after the informed consent had been signed and the aims of the study and confidentiality of their personal information were explained.

### Scientific trustworthiness of the results

In order to ensure the validity and truth of the data, we used Guba and Lincoln's four criteria of Credibility, Dependability, Confirmability, and Transferability [34]. To enhance the credibility of the findings, the participants were selected based on different groups of service provider teams, and sampling continued until data saturation. Also, the analysis results of the interviews were provided to the participants for confirmation. On the other hand, the review of data and results by the participants helped to identify the researchers' biases and remove them. Another way to increase the credibility of the data is to pay attention to the appropriate coverage of the data by themes and sub-themes, from which irrelevant data are removed and to which relevant data are included. Transferability of data was ensured by offering a comprehensive description of the subject, participants, data collection, and data analysis. Also, to increase the dependability of the research results, we used external check. The confirmability of findings was enhanced via investigator triangulation according to which more than one researcher is engaged in gathering, analyzing, and interpreting the data. The aim of investigator triangulation is to make the bias that may occur due to a single researcher’s fault less likely. The use of teamwork (investigator triangulation) reduced personal taste and controlled researchers' bias.

## Results

There were 30 interviewees in this study (24 users and 6 experts of SIB). The majority (*n* = 27) of the interviewees were female, and most of them were in the age range of 25 to 35 years and had a bachelor's degree. As far as work experience was concerned, 19 participants had 1–10 years of work experience and only 2 had managerial experience. Table 1 shows the demographic characteristics of the participants.

**Table 1:** Demographic characteristics of the interviewees

Demographic profile	Category	Number (Percent)
**Gender**	Female	27(90)
	Male	3(10)
**Age**	< 25	3(10)
	25–35	18(60)
	> 36	9(30)
**Educational attainment**	Bachelor’s degree	24(80)
	Master’s degree	3(10)
	General Practitioner	3(10)
**Work experience**	1–10	19(63.33)
	11–20	8(26.67)
	21–30	3(10)
**Managerial experience**	No experience	28(93.33)
	With experience	2(6.67)
**Job position**	Family health expert	12(40)
	Nutrition expert	3(10)
	Mental health expert	3(10)
	Midwife	3(10)
	Physician	3(10)
	System leader	1(3.33)
	Evaluation expert	3(10)
	Network development unit expert	1(3.33)
	IT expert	1(3.33)
	Total	30(100)

Totally, 42 components (24 components for benefits and 18 components for challenges) were extracted from the analysis. Three themes of Structure, Process, and Outcome were extracted from these components. The theme of Structure included four subthemes of “Financial resources”, “Human resources”, “Facilities”, and “Access to the Internet”; the theme of Process included three sub themes of “Training”, “Providing services” as well as “Time and workload”; the theme of Outcome included five subthemes of “Quality of health services”, “Access”, “Safety and personal distance”, “Screening and evaluation”, and “Research”. Extracted components and themes which highlighted benefits and challenges are presented in the following table.

In Table 2, components and sub-themes of the theme of Structure are classified based on benefits and challenges of SIB.

**Table 2:** Benefits and challenges of using SIB in health centers and frequency (%) of the participants who mentioned the statement (Theme of Structure)

Theme	subthemes	Components			
		Benefits	Frequency (%)	Challenges	Frequency (%)
Structure	Financial resources	Reducing expenses associated with paperwork, and printing, recording, correcting, evaluating, and retrieving information	27(90%)	Cost of purchasing and installing software and hardware, telecommunication costs, cost of converting paper documents into electronic records	21(70%)
				Cost of continuous training of personnel to work with the system and learn about the updated services of the system	12(40%)
		Improving the management of Health and usable drugs	9(30%)		
				The cost of replacing defective hardware and improving software	18(60%)
	Human resources	Improving management of human resources	18(60%)	Unwillingness of the personnel who do not have a positive attitude towards working with computers	7(23.3%)
				Lack of enough interest	10(33.3%)
		Providing services based on population	17(56.6%)	Reluctance of experienced users to work with the system (younger users were more willing to use the system)	12(40%)
	Facilities	Need of users to computers	5(16.6%)	Delayed delivery of support facilities and services	23(76.6%)
	Access to the Internet	Providing Internet access for all sections of the system	10(36.6%)	Internet disconnections during working hours	14(46.6%)

Many interviewees cited financial resources as a significant factor in implementing SIB. Reduced costs associated with elimination of paperwork was one of the identified benefits. “After the implementation of SIB in the centers, the costs related to paperwork significantly reduced” (Participant 7). One of the challenges related to financial resources was the cost of continuous training of staff. “Due to the novelty of this system, SIB users need training to work with the system, which imposes costs on the health sector” (Participant 12). Another challenge in establishing SIB was related to the human resources. “Personnel who did not have sufficient experience of working with a computer did not readily accept the system and did not have sufficient motivation to work with it” (Participant 23).

Table 3 shows the components and subthemes related to benefits and challenges of adopting SIB for the theme of Process.

**Table 3:** Benefits and challenges of adopting SIB in health centers and frequency (%) of the participants who mentioned the statement (Theme of Process)

Theme	subthemes	Components			
		Benefits	Frequency (%)	Challenges	Frequency (%)
Process	Training	Visibility of updated instruction for users to take care of individuals or record any type of health information (retraining the updated instructions while working with the system)	8(26.6%)	Lack of enough training classes, the length of classes, and method of teaching regarding coverage of services that SIB has to deliver	17(56.6%)
		Updating the programs, services and instructions of the Ministry of Health in the simplest possible way	4(13.3%)		
	Providing services	Importing the services in the system based on individual differences (i.e., age, gender, disease, pregnancy, etc.)	3(10%)	Incomplete definitions or lack of definitions for all services in the guidelines of the system	3(10%)
		Placing the updated services based on national health plans announced by the Ministry of Health	7(23.3%)		
				Incomplete or temporary recording of services due to loads of referrals and for accelerating the process	5(16.6%)
		Daily access to list of inquiries (daily) from all health centers and places affiliated to university after transferring the service provider	5(16.6%)		
	Time and workload	Saving time as there are not repetitive requests for information and statistics	12(40%)	Doctors’ limited time to use and learn how to work with the system	3(10%)
		No need to search and waste time to find information and health records of a patient	6(20%)		
				Doctors’ increased workload due to using this system	3(10%)

Some of the benefits and challenges that the interviewees expressed about this theme were related to time constraints and workload. They described the benefit of establishing SIB as follows: “We no longer need to search for information in the patient's health record in the archive, and we will have access to the information we need in a shorter time. This gives us more time to deal with other tasks (Participant 16). Some physicians stated that implementing SIB will increase their workload. “I am visiting and examining patients all the time and I do not have enough time to learn and use this system” (Participant 9).

Table 4 presents components and subthemes of benefits and challenges related to adopting SIB in health centers for the theme of Outcome.

**Table 4:** Benefits and challenges related to adopting SIB in health centers and frequency (%) of the participants who mentioned the statement (Theme of Outcome)

Theme	subthemes	Components			
		Benefits	Frequency (%)	Challenges	Frequency (%)
Outcome	Quality of health services	Decreased errors and increased quality by service providers due to smart delivery of services and illustrating guidelines particularly required actions	18(60%)	No modifiability of recording errors	10(33.3%)
	Access	Possibility of access to quality and planned services for all people	5(16.6%)	No interoperability of electronic records at different centers (excluding the university), increased errors, and repetitive processes (for not having patients' records)	3(10%)
		Possibility of access to integrated information for different users at any time and in any place and simplicity of information reporting and transmission	15(50%)		
	Safety and privacy	No possibility of missing patient health records	6(20%)	High accessibility of clinical data and exchange of information among different centers	10(33.3%)
	Screening and evaluation	The possibility to receive complete and integrated records related to activities of health units or health service providers at any time	3(10%)	No discrete access to doctors' care indexes, nutrition experts, and psychology experts at health posts due to their identified roles at health centers	2(6.6%)
		Removing the problem of illegibility and lack of access to paper records	2(6.6%)		
		Impossibility of changing the time recorded for service delivery	12(40%)		
		Impossibility of changing and amending information after 24 h	12(40%)		
		Providing a framework for documentation	10(33.3%)		
		Providing a dashboard of indices regarding the provider and health unit	4(13.3%)		
	Research	Providing a very proper information center for producing and managing medical knowledge	2(6.6%)	The possibility of fake recorded services in the system by users	12(40%)

Most of the interviewees mentioned the benefits and challenges with respect to security and privacy. “With the implementation of SIB, the availability of people's health information is enhanced, but it must be born in mind that this data is accessible from different centers, and this may endanger its confidentiality” (Participant 26). Some experts believed that one of the most important challenges in establishing SIB is the possibility of registering fake data in system. “We must be aware that the services recorded by users in the system may not be actually provided to individuals, so serious planning is needed in order to control more accurate recording of services” (Participant 14). One of the important benefits mentioned by the interviewees was screening and evaluation. “Because in the previous methods, different people were recording information in paper records, we always had the problem of illegible paper records, which led to careless provision of services and waste of time, but with the implementation of SIB, this problem was also solved” (Participant 29).

## Discussion

Nowadays, many countries dedicate a part of their electronic health strategic plans to the design and implementation of EHR as one of their priorities [35]. Developing countries like Iran have also made efforts in this field. Considering the importance of continuous monitoring of these systems and the need to know their strengths and weaknesses, in the present study, the benefits and challenges of adopting SIB were classified into three themes of Structure, Process and Outcome based on the views elicited from users and experts of SIB in health centers.

### SIB structure

The use of paper records is associated with problems such as increased costs of printing forms and folders [10]. One of the benefits identified in the theme of structure was the reduced costs of paper processes, which is in line with the results of Fakhrzad et al. [10]. In Ghayoomzade et al., reduced paperwork and coherent files were among the benefits mentioned by SIB users. They were very pleased to save paper and avoid cutting down more trees [36].

Most of the challenges related to human resources included lack of enough interest, rejection of the electronic system, and lack of incentives for users of the system. Users’ lack of sufficient interest should be carefully addressed because institutionalization and sustainability of EHR is almost impossible without the comprehensive participation of its users, and this sustainability is created when service providers are satisfied with SIB so that they can register services and information with sufficient interest [23]. Cho et al. showed that self-efficacy is the strongest factor affecting users' resistance to EHR adoption [37]. On the other hand, support from the government and insurance companies were among the significant facilitators suggested by other studies [38, 39]. In addition, older users and those who have little experience in working with computers were reported to be less willing to use EHRs. In another research, the satisfaction of SIB users had an inverse relationship with age [23]. Brumini et al. concluded that younger nurses had a positive attitude toward using EHRs [40].

Financial resources were another challenge cited by the respondents. In addition to telecommunication costs, most of the expenses were spent on buying and installing software and hardware and replacing paper documents by electronic records. Training the staff also calls for financial resources [41–44]. Furthermore, the secondary costs or the cost of permanent protection of the system include replacing defective hardware and upgrading software [41–45].

Another challenge identified by SIB users was the delay in delivery of facilities and support services. In other studies about SIB, SIB users were dissatisfied with lack of knowledge and inadequate response of SIB managers to questions regarding how to work with it [24] and the slow process of fixing the system's faults [27, 36]. In Abolghasemi et al., the component frequently emphasized by SIB users was the system support component [46]. The results of Tavakoli et al. also showed that the organizational and technical support of an information system will increase the motivation and willingness of users to use it [47]. Therefore, managers should pay more attention to providing timely and appropriate support services to SIB users.

Disconnection of the Internet during office hours was another major challenge identified in this study. The country's poor Internet infrastructure is one of the most important problems in provision of electronic health services in Iran. The SIB system has also been impaired or slowed down for this reason [3]. Enumerating the shortcomings in the communication infrastructure of Iran, Nasiripour et al. mentioned lack of proper telecommunication coverage and lack of Internet access in many parts of the country as the main barriers to the development of e-health [48]. Howard et al. and Laitinen et al. stated limited networks (Internet) as important barriers to successful EHR implementation [49, 50].

### SIB process

One of the most important benefits identified for SIB was the easy visibility of the updated instructions. In a similar research, it was stated that observing the instructions in SIB is very useful because it scientifically shows the next steps, and these instruction are followed, subjective decisions are reduced [36]. In the present study, users considered time saving as a benefit of SIB due to removing redundant demands for information and statistics. Furthermore, Shachak et al. found that EHRs lead to fast recovery of past records of the patients, and improving service delivery to them [51], and these results are in line with the findings in the present study.

In the present study, the users of the system pointed to the challenges related to the training problems of the Process theme. They believed the number of courses, their length, and method of teaching did not match the diversity of EHR services, which is consistent with the research results of Hazhir et al. [26]. It can be concluded that the courses offered to health care personnel to improve their skills of using electronic system do not seem to be enough, and EHR designers and implementers need to make a closer connection between electronic systems and the trained staff to improve the immature health system of Iran [10]. Previous studies have found that computer skills of users have an impact on both practicality and user-friendliness of EHRs [52]. This can be accomplished by providing proper and adequate training on the systems. Columbus reported that the average understanding and attitude of the participants in using EHRs was 58% and 64%, respectively before training, which rose to 72% and 78% after training. In other words, training health care providers is a vital factor in improving their readiness to use the system [53]. In this regard, some studies stated that the most important factor affecting the successful implementation of electronic systems in the health system is preparation and thorough familiarization of human resources [54].

One of the challenges raised in the current research was that some services were not defined in SIB. Similar to the current research, a previous study introduced lack of definition and impossibility of registering all services provided by health care providers as one of the most important challenges of SIB, which causes dissatisfaction and unwillingness of providers to perform activities [36].

Another challenge in the Process theme is the time-consuming nature and workload of the system. SIB users pointed out that the large number of service recipients and the time-consuming completion of service registration in the system led to incomplete registration of services, which is consistent with the results of Jafari et al. [3]. In addition, in a similar study, the most important reason for service providers' dissatisfaction was the large volume and number of SIB items [23]. There are many challenges for physicians as end users of EHR that limit the potential of this record to facilitate their work and improve the quality of patient care [55]. Whether the use of EHR for physicians improves efficiency or not is still considered controversial [56]. Doctors complained that they did not enough time to learn and use the system. The results of a research conducted by Sim and Miller reported that the time-consuming nature of using the system was among the disadvantages that led to reduced communication between health care providers and patients [57]. However, in Shield et al., doctors believed the EHRs help them reduce wasting of time [58], which is not consistent with the findings of this study. Redesigning or revising SIB based on the needs of service providers, especially physicians who face high workloads, can increase its efficiency [29].

### SIB outcome

Enhancing the quality of health services is one of the benefits of SIB as indicated by respondents. Similarly, in a study by Jebraeily et al., most positive views that the respondents held about adopting EHRs included increased quality of health services and improved documentation [59]. In another study, SIB's short message service for tracking and reminding prenatal care led to an increase in pregnant mothers’ satisfaction with the services provided [60]. The quality of vaccination services also increased. Following the results of some studies regarding the strength and proper performance of SIB in the field of vaccine registration [49], it can be argued that SIB during the prevalence of Covid-19 has also provided a suitable basis for vaccine registration and that this system can be very useful for rapid vaccination and thus taking a positive step towards controlling the disease. In other studies, the improvement of the quality of care after the adoption of EHR was stated [61–63].

One of the benefits of SIB was that it is not possible to edit information, which according to the participants, reduces the possibility of information distortion. Jafari et al. also listed the deletion-protection of recorded information and the ability to edit the care only up to the first 24 h as benefits of SIB [3]. In another related research, SIB users pointed to this benefit and believed that service providers should be able to have the necessary accuracy and be able to record information correctly in time [36].

The present study showed that the risk of patient health information loss is low and that SIB could lead to better documentation of the information. Previous studies have found that permanent retention of information, prevention of information loss, non-distortion of information [3], and improved documentation [36] were among the benefits of using SIB, which is in line with the results of the present study. According to the results of Moody et al. who investigated the nurses’ understanding, viewpoints, and preferences, 75% of nurses believed EHRs could improve documentation while 54% believed that with respect to confidentiality of information, the risk of EHRs is less than that of paper records. Furthermore, most of the nurses (81%) believed EHRs were “more of a help than hindrance to care” [64].

One of the important benefits identified in this study was quick and timely access to integrated information and information transfer. In this regard, the result of a similar study showed that integration and sharing of information using SIB is well possible and the availability of information in all health centers increases coordination in service delivery [26]. Gordon et al. also found that EHRs provide faster access to patient information for users by enabling data sharing [65]. Shahmoradi et al. introduced “quick and timely access to information” as the most important strength of EHR implementation from the point of view of managers [4].

Another benefit, according to the present study, was the simplification of the process of information report. Jafari et al. also mentioned the following as the main advantages of SIB: providing a general and fast report graphically and ease of communication with higher levels [3]. The results of another research also showed that the reporting feature has made good progress in improving SIB compared to the past [24]. The findings of Bitaraf et al. also showed that the provision of detailed reports was one of the factors influencing the satisfaction of SIB users [25]. Contrary to the results of the present study, it was reported in another study that SIB users raised problems such as the inconsistency between the deputy's report and the comprehensive health service center's report and failure in center-specific extraction of data from vital horoscope for reporting [36]. SIB made possible providing fast reporting of health information for providers as it removed paperwork and facilitated information transfer. Therefore, managers can have access to facts and figures of their area and consider screening of their staff [26]. As Miller and Sim reported, exchange of information via EHRs has several benefits including elimination of paper-based and parallel reports and making it possible for users to experience ubiquitous and easy reporting of information [57].

Confidentiality and privacy of information discussed under the theme of Outcome was another concern voiced by the users and evaluators working with the EHR system. In a similar study, the possibility of information loss was the main concern of EHRs users [66]. The confidentiality of the records is threatened by the fact that all staff can access the information and that there are hackers and perpetrators who may violate this confidentiality [10]. Hence, there is an urgent need for healthcare organizations to find strategies to help secure the EHR [67]. Also, in order to ensure information security and legal follow-up in case of disruption, appropriate legal infrastructure is needed [3]. The study of Farzandipour et al. shows that Iran does not have comprehensive requirements regarding the safety of electronic health record information, and using the experiences of successful countries in this regard is effective [68]. Addressing EHR security and privacy challenges, Keshta & Odeh recommended that an efficient encryption scheme that can be easily applied by healthcare professionals be implemented in the latest EHR revisions [67].

Another challenge identified for SIB was the possibility of registering fake services. This challenge of SIB was also mentioned in a similar study which found that payment based on services sometimes leads to registration of fake, duplicate or unnecessary services in SIB [36]. In a study on the quality of SIB information recording, Gharaei et al. stated that the quality of this information may be compromised due to the dependence of part of the employees' income on the quality of information recording [69].

Considering that the current application of SIB which is mostly used for recording information and services, researchers are advised to study the possibility of developing a virtual network to provide services. In addition, a qualitative study can be conducted using the opinions of SIB designers and managers about the capabilities of this system to support the decision. Considering the identified challenges for SIB, it is suggested to conduct studies aimed at investigating the effectiveness and applicability of a large amount of information in order to solve health problems and to compare the various plans available to increase the confidentiality of information.

## Limitations

First, our study was limited to only three populations. It is necessary to use the opinions of more users and experts from different areas in Iran to evaluate benefits and challenges of SIB. As a consequence, care should be applied when generalizing the findings. In addition, it was difficult to conduct interviews with some SIB users and experts due to their high workload caused by the Covid-19 pandemic. To address this limitation, we arranged interviews with participant on several occasions.

## Conclusion

In the present study, the benefits and challenges of SIB were examined in three themes: structure, process, and outcome. In theme of structure, reduction of costs related to paper processes and improving human resource management were the main benefits of SIB while lack of sufficient motivation among employees, the costs of converting the paper system into electronic system, and Internet disconnections during office hours were its major challenges. The main benefit of SIB under the theme of process was time saving while its challenges were problems associated with training and increased workload. In theme of outcome, the benefits of SIB included increased quality of services and rapid reporting of information while the possibility of violation of information confidentiality was the main challenge identified for SIB. Most of the identified benefits were related to the theme of outcome, and most of the identified challenges were associated with the theme of structure. In order to solve the identified SIB challenges, it is necessary to pay attention to the following points: using appropriate reward systems to increase people's motivation, comprehensive planning for step-by-step training of SIB users, correct budgeting based on conditions and facilities, using the experiences of other successful countries, and creating appropriate legal infrastructure to increase information security and confidentiality. The results of the present study can help countries that have not yet launched systems like SIB to take the necessary steps to build their own system with minimal challenges.

## Data Availability

The datasets analysed during the current study available from the corresponding author on reasonable request.
